# Synthesis and Characterization of Zeolites Produced from Low-Quality Coal Fly Ash and Wet Flue Gas Desulphurization Wastewater

**DOI:** 10.3390/ma14061558

**Published:** 2021-03-22

**Authors:** Paulina Nowak, Barbara Muir, Agnieszka Solińska, Małgorzata Franus, Tomasz Bajda

**Affiliations:** 1 PGE Energia Ciepła S.A., Department of Research and Development, ul. Ciepłownicza 1, 31-587 Kraków, Poland; 2 Faculty of Geology, Geophysics and Environmental Protection, al., AGH University of Science and Technology, Mickiewicza 30, 30-059 Kraków, Poland; muir@agh.edu.pl (B.M.); asolinska@agh.edu.pl (A.S.); bajda@agh.edu.pl (T.B.); 3 Department of Construction, Faculty of Civil Engineering and Architecture, Lublin University of Technology, Nadbystrzycka 40, 20-618 Lublin, Poland

**Keywords:** zeolite, fly ash, low-temperature synthesis, microporous materials, sorption properties

## Abstract

This study investigated a low-energy-consuming procedure for the synthesis of zeolite materials from coal fly ash (CFA). Materials containing zeolite phases, namely Na–X, Na–P1, and zeolite A, were produced from F–class fly ash, using NaOH dissolved in distilled water or in wastewater obtained from the wet flue gas desulphurization process, under atmospheric pressure at a temperature below 70 °C. The influence of temperature, exposure time, and alkaline solution concentration on the synthesized materials was tested. In addition, chemical, mineralogical, and textural properties of the obtained materials were determined by X-ray diffraction (XRD), X-ray fluorescence (XRF), scanning electron microscopy (SEM), and cation exchange capacity (CEC). Cd(II), Ni(II), NH_4_^+^ cation, and Se(VI) anion sorption experiments were conducted to compare the sorption properties of the produced synthetic zeolites with those of the commercially available ones. Zeolitization resulted in an increase of CEC (up to 30 meq/100 g) compared to raw CFA and enhanced the ability of the material to adsorb the chosen ions. The obtained synthetic zeolites showed comparable or greater sorption properties than natural clinoptilolite and synthetic Na–P1. They were also capable of simultaneously removing cationic and anionic compounds. The structural, morphological, and textural properties of the final product indicated that it could potentially be used as an adsorbent for various types of environmental pollutants.

## 1. Introduction

Zeolites are microporous, crystalline hydrated aluminosilicates composed of tetrahedral SiO_4_ and AlO_4_^−^ units [1]. Their essential structural feature is a three-dimensional tetrahedral framework, in which each oxygen atom is shared by two tetrahedra. Some Si^4+^ ions are substituted by Al^3+^ ions in the framework. This results in a net negative charge arising from the difference in valency between the (AlO_4_)^5−^ and (SiO_4_)^4−^ tetrahedra and normally located on one of the oxygen anions linked to an aluminum cation [2]. Therefore, the negative sites formed are balanced by counterions, which in most cases are alkali or alkaline earth metals, such as Na^+^, K^+^ or Ca^2+^ [3].

The structure of zeolites is characterized by a regular construction of channels and chambers, which confers them a number of unique qualities including large sorption capacity as well as ion-exchange, molecular-sieve, and catalytic properties. Due to these features, zeolites have gained a great deal of attention from researchers. Moreover, characteristics such as porosity, surface area, and chemical resistance are given significant importance in industries, and therefore, zeolites have a high potential for wide application in the areas such as removing undesirable heavy metal ions from industrial effluent waters [4], environmental remediation [5,6] and water treatment [7], soil construction and repair, soil amendment, animal nutrition and health, aquaculture, building materials, heat storage and solar refrigeration [8], catalysts and molecular sieves [9], etc. Most natural zeolites are formed as a result of volcanic activities, while synthetic zeolites result from the reactions of alkaline medium with: Sodium silicate and sodium aluminate or by-products of coal combustion [10,11]. The demand for rare and specific minerals, such as zeolites, has been increasing in recent years, and thus, finding a low-cost and effective method for their synthesis remains a challenging issue.

Coal fly ash (CFA) is produced during solid fuel combustion carried out for the production of electricity and heat. It is captured from flue gas in electrostatic precipitators or filter bags as fine dust consisting of glassy spheres [12]. The quality of CFA is determined by many factors such as fuel quality, combustion temperature, air-to-fuel ratio, the size of coal particles, combustion rate, and the type of installations used in the combustion process [13]. The chemical composition of CFAs resulting from hard coal combustion comprises mostly silicon, aluminum, and iron in the form of oxides. Quartz, mullite, hematite, and magnetite are the most frequently occurring crystalline phase [14], which makes CFAs similar to volcanic ashes, the precursors of natural zeolites. CFA has been recognized as a good substrate for the production of synthetic zeolites and synthesis conditions have been widely discussed in the literature [15,16]. According to the available data, CFA can be used for direct hydrothermal synthesis of zeolites [17], microwave assisted hydrothermal synthesis [18], hydrothermal synthesis of zeolites from alkali-fused CFA [19] or hydrothermal synthesis of zeolites using extracted silica or mine waters [20]. The main area in which CFA, originating from hard coal combustion, is utilized is the construction industry, where it is used as a substrate for the production of cement, concrete, and aggregate [21]. However, due to the implementation of new pro–ecological installations, mainly NO_x_ emission control (deNO_x_), in the power and heat production facilities, the quality of CFA is subject to change, which can make it impossible to be applied in the construction industry. Primary deNO_x_ installations affect the total organic carbon (TOC) in CFA, and secondary deNO_x_ installations can lead to ammonia slip and ammonia contamination of the final product [22]. CFA that does not meet the requirements of the construction industry, is harder to be utilized, and in many cases, it must be stored in landfills, causing a considerable environmental impact [23,24]. To ensure a sustainable future for the environment and local ecosystem, it is particularly important to find a way to prevent the storage of industrial wastes and to recycle and reuse them [25]. Many of the approaches proposed for the recycling and reuse of energy-sector wastes are not economic. Thus, significant research efforts are required to develop cost-effective recycling and reuse options and to prevent the migration of contaminants from rehabilitated waste repositories in the long term [26].

Furthermore, due to the aggravating emission norms, most of the large-scale power plants have been equipped with different kinds of flue gas desulphurization (FGD) installations in recent years. Among the technologies used, wet flue gas desulphurization (WFGD) is the most popular, which gives a considerable amount of gypsum and wastewaters as by-products [27]. The generated wastewaters are chemically cleaned in sewage treatment plants, and after reaching the required chemical composition, disposed into rivers [28]. The commonly used wet scrubber systems generate wastewaters containing a high concentration of ions, heavy metals, total suspended solids, and ammonia as a consequence of ammonia slip and hence, requiring treatment before disposal [29]. Wastewater plants set up for WFGD installations are usually one- or two-stage systems, in which the main methods of purification are precipitation and sedimentation. The reagents added in the treatment process, including trimercaptotriazine (TMT-15), iron coagulates (PIX), and aluminum coagulates (PAX), lead to the precipitation of heavy metals in a sparingly soluble form (hydroxides, sulphides or organic sulphide complexes) [30]. The largest amount of calcium hydroxide Ca(OH)_2_ is added to precipitate SO_4_^2−^ sulphates, which also contributes to the precipitation of some metal cations in the form of hydroxides. The composition of WFGD wastewater is affected by many variables such as coal and limestone composition, type of scrubber, and the gypsum-dewatering system used. Coal contributes to the presence of acidic gases (chlorides, fluorides, and sulphates), as well as volatile metals, including arsenic, mercury, selenium, boron, cadmium, and zinc, while limestone contributes to the presence of iron and aluminum (formed from clay minerals) in the WFGD wastewater. The other common contamination is caused by the build-up of chlorides, which are typically limited to 10,000–12,000 mg/L. Due to the new regulations on emission levels, there is a high demand for new wastewater cleaning technologies that could be used in large-scale facilities. Due to their high sorption properties, zeolites are one of the possible solutions. However, due to difficulties in process scaling and the high cost of raw materials, they are not used so far. Considerable interest is also shown in reducing the amount of disposed sewages due to growing environmental costs [31].

To meet the increasing demand for synthetic, high–quality, and low–cost zeolite materials that can be used in the energy sector, a new energy-efficient technology for zeolite production has been proposed. This approach is based on the production of these functional materials from low-quality CFA, at a low temperature using wastewater obtained from WFGD installation rather than chemically cleaned water, and is thus consistent with the Circular Economy policy.

## 2. Materials

### 2.1. Fly Ash

**Figure 1:**
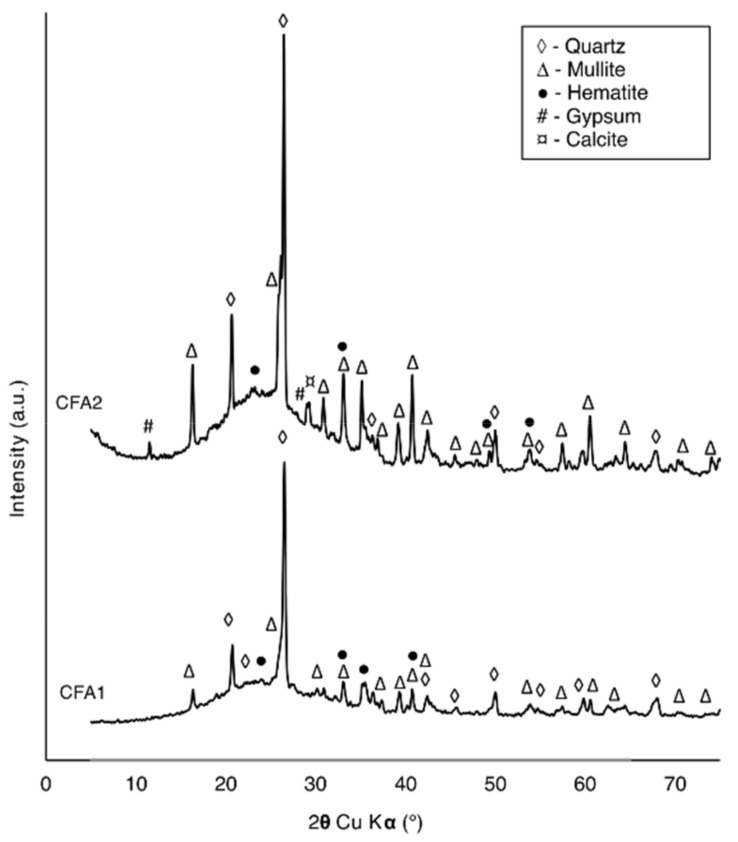
XRD patterns of the first raw coal fly ash (CFA1) and second (CFA2).

Two FA samples were used in this study (CFA1, CFA2), both obtained as a by-product of the hard coal combustion process in a combined heat and power facility equipped with boilers operating with primary and secondary deNO_x_ installations. Different raw CFA (RCFA) samples were used in the first (CFA1) and second (CFA2) stage of the study. The tests were carried out in cooperation with the power-producing company, as part of the project aiming to find new ways for utilizing non quality coal fly ash. Since the Company is running few producing facilities, for the purpose of the original project, it was important to prove that the proposed zeolite synthesis method is viable for different raw fly ash parameters, and the results are resistant to its changes. Experiments were conducted in two separate stages with different objectives for each one of them. Therefore, two different samples were not an issue and they helped check whether the obtained results are comparable for different raw fly ash compositions. The raw CFA samples used in the study were a typical F-class ash consisting of aluminosilicate glass, mullite, quartz, and hematite as major components and gypsum and calcite in minor amounts (Figure 1). The chemical composition of CFA1 and CFA2 is presented in Table 1. Samples also contained ammonia (over 300 mg/kg). CFA contaminated with a high concentration of unburned carbon and ammonia can be categorized as low quality with limited utilization possibilities.

### 2.2. Wastewater from the WFGD Process

In the experiments, chemically cleaned wastewater obtained from the WFGD installation was used. The sewage came from a conventional power plant in which hard coal was used as fuel. The wastewater was characterized by high salinity and pH, which makes the gypsum (occurs in the form of Ca^2+^ and SO_4_^2−^ ions) more soluble. It contained unreacted calcium carbonate and trace amounts of combustion by-products. In addition, significant amounts of nitrogen compounds in various forms were observed (Table 2). Their presence can be attributed to the phenomenon of ammonia slip, which occurs as a result of nonselective noncatalytic reduction of NOx. Unreacted NH_3_ gets into the sewage, where it dissolves into NH_4_^+^ ammonium ions or is oxidized to NO_3_^−^/NO_2_^−^ anions. The range and average of the most important WFGD wastewater parameters are summarized in Table 2. The level of some metals was also determined, and their concentrations did not exceed the following limits (mg/L): Cd—0.0005, Fe—0.008, Mn—1.96, Ni—0.026, and Zn—0.031. The WFGD wastewater samples used in the experiments were picked freshly from the wastewater stream on the side of the WFGD installation.

### 2.3. Clinoptilolite and Zeolite Na–P1

In order to compare with the well-characterized natural and synthetic zeolites, the properties of the newly synthesized materials were compared with naturally occurring clinoptilolite and the synthetic zeolite Na–P1 described in the literature. A clinoptilolite-rich rock (Cp) was obtained from the deposit in Nižný Hrabovec, Slovakia (commercially available; ZeoCem, Košice, Slovenská), and synthetic zeolite Na–P1 was produced from F-class CFA by the hydrothermal conversion reaction, as presented by Franus (2012) [10] and Wdowin et al. (2014) [11]. Both materials have been described in the previous studies as sorbents of metals and organic compounds, Cp was characterized by Wołowiec et al. (2017) [32] and synthetic zeolite Na–P1 by Bandura et al. (2015) [33] and Szala et al. (2015) [34]. Prior to the experiments, all the samples were dried at 100 °C to remove the adsorbed water and stored in a desiccator until further use. All the test results were repeated until two identical indications in a row were obtained.

Cp is rich in clinoptilolite (~84%) with admixtures of K-feldspar, mullite, and quartz with a chemical composition [32], as presented in Table 1. The adsorption/desorption isotherm of Cp is classified as type II with the H3 hysteresis loop, which indicates the homogeneous distribution of mesopores and the presence of slit-shaped pores [35,36]. The particle size of Cp is bimodal with peaks located at 30 and 300 μm, which constitute 25% and 21% of the volume, respectively. The specific surface area of Cp (S_BET_) is 30.31 m^2^/g and the total volume of pores is 0.123 cm^3^/g [32].

Na–P1 used in this study is rich in the Na–P1 phase (~81%) with admixtures of quartz and mullite [33,34]. Its chemical composition is presented in Table 1. The adsorption/desorption isotherm obtained for Na–P1 has a composite of type II and IV isotherms. The Na–P1 hysteresis loop has H2 and H3 types, which suggests the presence of slit-shaped pores (plates or faces/edges, particles such as cubes) of nonuniform sizes or shapes or ink bottle-shaped pores. The distribution of Na–P1 particle size is homogenous with peaks at 20 μm. Its BET is 74.91 m^2^/g, and the total volume of pores is 0.225 cm^3^/g [33,37].

### 2.4. Methodology of Synthesis

The low-temperature zeolite synthesis was carried out in two stages. The first stage aimed to determine if it is possible to achieve zeolite synthesis from the low-quality CFA at a low temperature and with the use of WFGD wastewater. Once this was confirmed, the second stage proceeded in which the impact of the process parameters and the used medium on the composition and characteristics of the zeolite phase was tested. The unburnt carbon along with other volatile materials present in CFA was not removed from the samples prior to the synthesis. Samples were not ground or used for other preparations. The solid-to-liquid ratio was chosen based on previous studies [34]. All the chemicals used in this study were of analytical grade.

#### 2.4.1. First stage of the experiment

This stage consisted of four sets of experiments. In each batch, 65 g of raw CFA1 was mixed with (1) distilled water (DW), (2) WFGD wastewater (WW), (3) 3 M NaOH solution in distilled water (NaOH _(DW)_), and (4) 3 M NaOH solution in wastewater (NaOH _(WW)_), respectively (Table 3). During the synthesis, the samples were mixed on a magnetic stirrer for the first 24 h, and then placed in polyethylene terephthalate (PET) bottles. All the experiments were conducted at room temperature (average 24 °C), except for the first hour when the temperature was elevated to a maximum of 65 °C, due to the exothermic effect of dissolving NaOH. Samples were collected after 1, 3, 7, 14, 21, and 28 days, and later randomly until 31 weeks of synthesis. Experiments conducted in distilled water and wastewater solutions verified if CFA reacted with the used solvent itself.

#### 2.4.2. Second Stage of the Experiment

This stage was carried out in two rounds, with four sets of experiments each. In each set, 20 g of CFA2 was mixed with 400 mL of NaOH _(DW)_, placed on a magnetic stirrer for 24 h, and then transferred to PET bottles.

In the first round, synthesis solutions were heated at 65 °C for different times: (1) 0 h, (2) 1 h, (3) 12 h, and (4) 24 h (Table 3). The temperature used in this experiment could be easily and economically achieved in a power plant with the use of waste heat. A lot of waste heat is generated as a by-product of heat and energy production. It can be used to heat up wastewater, naturally occurring in a temperature up to 50 °C.

In the second round, CFA2 samples were treated with NaOH _(DW)_ solutions of various concentrations: (1) 3 M, (2) 4 M, (3) 5 M, and (4) 6 M (Table 3), but the solutions were not heated. For both rounds, samples were collected after 0.5, 1, 7, 14, and 28 days of synthesis.

## 3. Analytical Methods

All the collected samples were examined by X-ray diffraction (XRD) to analyze the existence of the synthetic zeolite phase, additionally porosity was also determined for all the samples from stage two of the experiment. Based on the obtained results, the synthetic zeolite (SZ) sample was chosen to evaluate the microstructure, chemical composition, porous texture, and sorption capabilities.

### 3.1. XRD and XRF

The mineral composition of the samples was determined by XRD using a X’pert PROMPD diffractometer with PW 3050/60 goniometer and a Rigaku SmartLab diffractometer (Tokyo, Japan) both with CuKα radiation and graphite monochromator. The measurements were conducted in the 2θ range from 2 to 65 or 75° with a step size of 0.05°. Diffraction data were processed with X’Pert Highscore Plus and XRAYAN software based on the International Centre for Diffraction Data (ICDD) database. The Rietveld refinements method [38] was used for the quantitative phase analysis of the samples.

The chemical composition of the samples was determined by X-ray fluorescence (XRF) with a wavelength-dispersive XRF spectrometer ZSX Primus II RIGAKU (WD-XRF ZSX Primus II Rigaku, Tokyo, Japan) with Rh anode (4.0 kW). The elements analyzed ranged from fluorine to uranium, and the detection limit for each of these ranged between 0.01% and 100%. Based on the scan results, a semiquantitative analysis was performed with the SQX Calculation software using the fundamental parameter method. Prior to the XRF analyses, LOI was determined by heating the samples to 950 °C and calculating the weight loss occurring during the heating process. The amount of each element was normalized up to 100% considering the LOI value. To measure the content of carbon (C) an automatic analyzer (EA 1108 Carlo Erba Instruments, Strada Rivoltana, Rodano, Milan, Italy) was used.

### 3.2. Textural Analysis

CEC was determined by the Ba-Mg ions adsorption/desorption method [39]. BET and the porosity of the samples were analyzed using the N_2_ gas adsorption/desorption isotherms at 77 K with an ASAP 2020 apparatus (Micromeritics, Norcross, GA, USA). The samples were outgassed for 12 h at 378 K. The BET equation was used for calculating the specific surface area (*S*_BET_) [40]. The total pore volume (*V*_tot_^0.99^) was estimated from the amount of N_2_ adsorbed at a relative vapor pressure (*P*/*P*_0_) of ∼0.99. The volume of micropores (*V*_mic_^DR^) was calculated using the Dubinin–Radushkevich method [41]. The mesopore volume (*V*_mes_^BJH^) was determined from the adsorption branch of the isotherms using the Barrett–Joyner–Halenda (BJH) method [42] in the mesopore range proposed by Dubinin (1960) [41]. The macropore volume (*V*_mac_) was calculated using the following equation:

*V*_*mac*_ = *V*_*tot*_^0:99^ – (*V*_*mic*_^*T*^ + *V*_*mes*_^*BJH*^) (1)

Air-dried, uncoated samples were examined by scanning electron microscopy (SEM) using a variable pressure field-emission scanning electron microscope (FEI Quanta 200, FEI, Graz, Austria) equipped with an energy-dispersive spectrometer for elemental microanalysis.

### 3.3. Multi-Element Sorption Experiments

To determine the sorption capacity of the products resulting from the reaction of CFA with reagent solutions, the obtained sample (SZ) was compared with Cp and Na–P1. The SZ was collected after 12 h of synthesis in the second stage of the experiment, for the set in which the synthesis solution was heated at 65 °C for 12 h. The sample was rich in zeolite A and Na–P1. To determine the sorption properties, experiments involving the simultaneous removal of cadmium Cd(II), nickel Ni(II), selenium Se(VI), and ammonium NH_4_^+^ ions were performed. These elements were chosen since they are difficult to be removed from WFGD by standard techniques, and specific requirements are defined by the European and national environmental law.

The batch sorption experiments were performed in duplicates for three types of sorbents: Cp, Na–P1, and SZ obtained in the present study. All the experiments were carried out at room temperature (22 ± 2 °C), from multi-element solutions with initial concentrations (C_0_) of:

- 1 mg Cd(II)/L + 1 mg Ni(II)/L + 1 mg Se(VI)/L + 100 mg NH_4_^+^/L; initial pH of solution (pH_in_) = 6;
- 2 mg Cd(II)/L + 2 mg Ni(II)/L + 2 mg Se(VI)/L + 200 mg NH_4_^+^/L; pH_in_ = 5.96; and;
- 5 mg Cd(II)/L + 5 mg Ni(II)/L + 5 mg Se(VI)/L + 300 mg NH_4_^+^/L; pH_in_ = 5.81.

Each multi-element sorption solution was prepared by dissolving Cd(Cl)_2_ 2.5 H_2_O, Ni(NO_3_)_2_ 6 H_2_O, Na_2_SeO_4_, and NH_4_Cl, respectively, in redistilled water. In all the experiments, 250 mg of Cp, Na-P1 or SZ was placed in 50 mL Falcon tubes, and 25 mL of one of the three multi-element solutions was added (solid-to-liquid ratio of 10 g/L). The resulting mixtures were shaken for 24 h, centrifuged at 4700 rpm for 10 min, and decanted. After the sorption experiments, the equilibrium pH (pH_eq_) was measured.

The concentrations of Cd(II) and Ni(II) in solutions were analyzed by atomic absorption spectroscopy using a SavantAA spectrometer (Savant AA GBC Scientific Equipment, Braeside, Australia). The concentration of Se(VI) was analyzed by inductively coupled plasma optical emission spectrometry using a Perkin Elmer Optima 7300 DV spectrometer (Mettler Toledo, Columbus, OH, USA). The concentration of NH_4_^+^ was analyzed by Nessler’s colorimetric method [43] using a UV-Vis Hitachi U-1800 spectrophotometer (Hitachi, Tokyo, Japan). The adsorption capacity *S* (mg/kg) was calculated as follows: Savant AA GBC Scientific Equipment, Braeside, Australia

(2) $$S=100\left(\frac{C_{0}-C_{eq}}{C_{0}}\right)$$

where C_0_ is the initial concentration of Cd(II), Ni(II), Se(VI) or NH_4_^+^ (mg/L) and C*_eq_* is the concentration of Cd(II), Ni(II), Se(VI) or NH_4_^+^ in the equilibrium solution (mg/L) after adsorption.

## 4. Results and Discussion

### 4.1. Phase and Chemical Analysis

**Figure 2:**
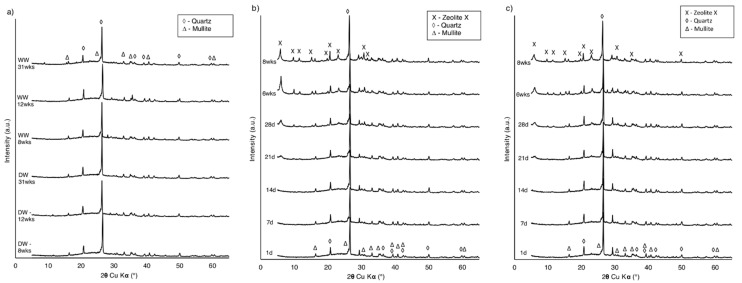
XRD patterns of samples from the first stage of the experiment: (**a**) CFA + distilled water (DW) and CFA + waste water (WW); (**b**) 3 M NaOH (DW); and (**c**) 3 M NaOH (WW).

**Figure 3:**
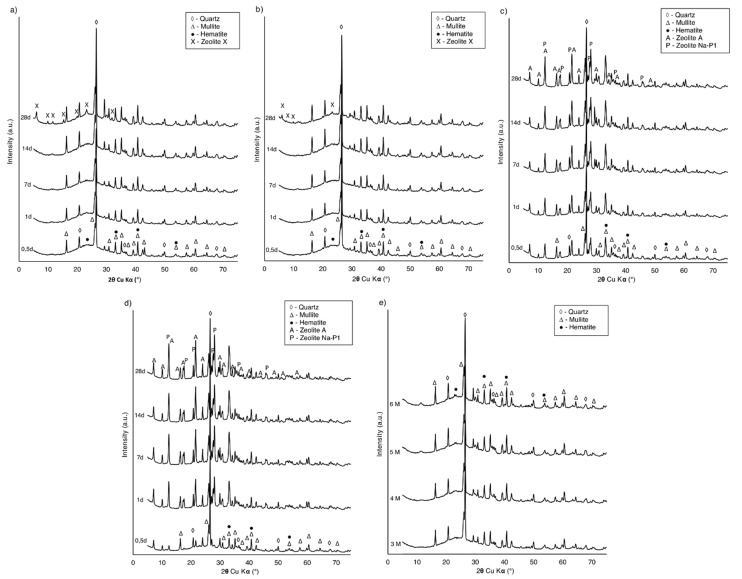
XRD patterns of samples from the second stage of the experiment with 3 M NaOH (DW): (**a**) No heating; (**b**) 1-h heating; (**c**) 12-h heating; (**d**) 24-h heating; and (**e**) after 4 weeks of synthesis with 3–6 M NaOH (DW).

The typical pH spectrum observed during zeolite synthesis experiments (when the NaOH solution was applied), ranged from 13 to 13.3. This is a typical value for hydrothermal zeolite synthesis from coal fly ash [44,45]. To determine whether it is possible to synthesize zeolite from low-quality CFA under the chosen conditions, all the collected samples were subjected to XRD measurements. The diffraction patterns for the first and second stages of the experiment are presented in Figure 2 and Figure 3, respectively.

For samples from the series prepared with distilled water and wastewater, no zeolite crystallization was observed after up to 31 weeks of experiment (Figure 2a). The results indicated that these samples were composed mainly of quartz, which was represented by a set of characteristic diffraction peaks at 3.34, 4.25, 1.81, 1.54, 2.45, 2.28, and 2.12 d_hkl_ values (26.65, 20.85, 50.61, 59.95, 36.54, 39.45, and 42.46 2θ, respectively). In addition, less intense peaks were identified as mullite at 3.39, 3.42, 5.39, 2.20, 2.54, 2.69, and 1.52 d_hkl_ (26.29, 26.05, 16.48, 41.03, 35.34, 33.31, and 60.82 2θ, respectively) and as hematite at 2.70 and 2.51 d_hkl_ (33.11 and 35.61 2θ, respectively). A significant increase of the background in the 2ϴ angular range of 15–40° suggested the presence of amorphous aluminosilicate glass. XRD patterns were very similar to the raw CFA diffractograms, which proved the absence of CFA–solvent reactivity. Patterns of the materials collected from experiments with 3 M NaOH (distilled water/wastewater solutions) indicated that except for quartz, mullite, and hematite, the zeolite phase was present (Figure 2b). The synthesized products matched the characteristic peaks of zeolite Na–X (zeolite X) at 14.49, 4.24, 2.89, 3.83, 8.87, 7.57, and 5.76 d_hkl_ (6.10, 20.94, 30.85, 23.24, 9.97, 11.69, and 15.39 2θ, respectively), when compared with the XRD standard pattern of zeolite X from the PDF-2 release of the 2010 database formalized by the ICDD and the IZA-SC Database of Zeolite Structures. The first weak peaks corresponding to zeolite X appeared after 3 weeks of synthesis. In subsequent samples, an increase in the intensity of characteristic peaks was observed, reflecting the increasing amount of zeolite X and progress of the zeolitization process. Simultaneously, the background decreased, which proved that the components of the amorphous phase were getting converted into a crystalline phase. The zeolite phase appeared much sooner than expected, compared to the experiment of low-temperature zeolite synthesis conducted by Derkowski et al. (2007) [46]. Previous research proved that it is possible to synthesize zeolite from CFA, at a low temperature, without any prior treatment [46], although several months of storage of CFA in the NaOH solution was required. In the present study, the increase in temperature to 65 °C, during the first hour of the experiment, is most probably responsible for speeding up the zeolitization process. Similar results were also reported in the case of treating biomass fly ash with alkaline activators in 60 °C, though higher concentrations were used [47]. The type of zeolite obtained in the experiment is also comparable with another study, in which zeolite X was synthesized at a low temperature from CFA with seawater [48]. Zeolite X was present in the samples prepared from both distilled water and wastewater NaOH solutions (Figure 2b,c), however, the XRD patterns indicated that the synthesis was more efficient in distilled water. This can be explained by the complex chemical composition of wastewater [49] and high Cl^−^ ion concentration. As proven in previous research [46], crystallization of the zeolite phase is favored by the absence of Cl^−^ ions in the solution.

Analyzing the XRD data from the second stage of the experiment, in sets where the temperature was applied with different durations, the first peaks corresponding to the zeolite phase started to appear at different rates, depending on the heating time. For samples with up to 1 h of heating, the diffraction peaks representing zeolite X (Figure 3a,b) were observed after 2 weeks of zeolitization with no other neo-formed mineral found. This is consistent with the results obtained in the first stage of the experiment. For samples with 12 and 24 h of heating time, the first peaks corresponding to the zeolite phase appeared after 12 h of the experiment (Figure 3c,d). The zeolite phase was recognized by the characteristic interplanar distances: d_hkl_ = 7.10, 5.02, 4.10, and 2.68 (12.46, 17.66, 21.67, and 33.38 2θ, respectively) for Na–P1 and d_hkl_ = 7.10, 5.02, 4.16, and 2.98 (12.46, 17.65, 21.36, and 29.94 2 θ, respectively) for zeolite A. For all the conditions, an increase in the intensity of characteristic peaks at subsequent samples was observed, indicating further progress of the zeolitization process. Depending on the reaction conditions, different and mixed products of synthesis are described in numerous reports [49,50]. Extending the heating time was identified as a sufficient factor to change the dominant zeolite phase in the collected samples, as well as to speed up the zeolitization process.

The zeolite phase was absent in the material collected from the experiments in which various concentrations of sodium hydroxide were tested (Figure 3e). Mullite and quartz occurred in amounts that did not significantly differ from the contents in raw CFA. The amorphous substance was noticed due to the rise of the background line in the XRD pattern. Many factors affected the type and crystallinity of synthetic zeolite formed from CFA during hydrothermal processes [49], including treatment time, temperature, chemical composition of raw ash, type of alkaline hydroxide, alkaline concentration, and liquid-to-solid ratio [13]. The results obtained in the experiment proved that even a minor temperature applied for a limited amount of time is a crucial factor for zeolitization from raw CFA, regardless of the applied alkali concentration.

Based on the XRD pattern analysis and the aim of the work, a representative sample of SZ resulting from the experiments was chosen for further tests. It was decided that the investigation should be carried out for materials collected after 12 h of synthesis from the second stage of the experiment, set with a 12-h heating time. This is the shortest time, with the smallest energy input, after which the zeolite phase was observed in a significant amount. The SZ sample is a mixture of zeolite A and zeolite Na–P1 (Figure 3c). From the analyzed XRD patterns, surface parameters and sorption capabilities measured for this sample are expected to be intensified for subsequent samples, as well as for samples with a longer heating time. The experiment duration would also be sufficient for use in a large-scale installation at the power plant facilities. Using the Rietveld refinements method quantitative phase analysis shown in the SZ sample: Zeolite A–31%, zeolite P–25%, mullite–17%, quartz–9%, aluminosilicate glass–18%.

The chemical composition of the collected SZ sample was as follows (wt.%): SiO_2_—33.24, TiO_2_—1.17, Al_2_O_3_—29.26, Fe_2_O_3_—5.38, CaO—2.68, MgO—2.16, Na_2_O—8.22, and K_2_O—0.62, LOI—19.41. The main constituents of zeolite were Si and Al, with a visible contribution of sodium. This is very close to the chemical composition of synthetic zeolite Na–P1 used as a comparative material.

### 4.2. Textural Characteristics

**Figure 4:**
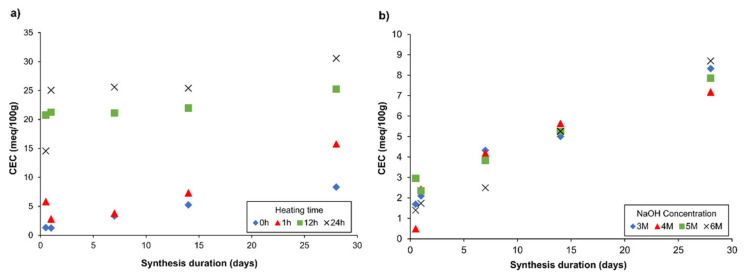
Influence of (**a**) heating time; (**b**) NaOH (DW) concentration on the cation exchange capacity (CEC) parameter.

The CEC of each sample, collected from stage two of the experiment, showed increasing values in reference to the synthesis duration, temperature, and NaOH concentration (Figure 4). As the Si/Al ratio is represented by the CEC value, it is a good reference example, in which the influence of synthesis temperature, NaOH concentration, and time can be determined. It was observed that increasing the concentration of NaOH did not influence CEC to a great extent (Figure 4b). However, running the synthesis process at an elevated temperature for a certain period of time significantly speeds up the appearance of the synthetic zeolite phase, as reflected by the CEC value (Figure 4a). For example, the CEC values of the materials synthesized at 65 °C for the first 1, 12, and 24 h and later stored for 14 days at room temperature were 7.32, 21.99, and 25.40 meq/100 g, respectively. The CEC value of the material synthesized for 14 days without extra heating was only 5.26 meq/100 g. From the obtained results, it can be concluded that temperature is a critical parameter for the synthesis of the zeolite phase.

Considering the most accurate process conditions for industrialization (CEC vs. synthesis time and consumed energy), the sample SZ collected after 12 h of heating at an elevated temperature of 65 °C was chosen for further consideration and textural analysis. The results obtained for synthetic zeolite were compared with those of reference materials (Cp and Na–P1) (Table 4). Cp was characterized by the highest CEC value (40.69 meq/100 g) and a high share of micropores and mesopores (90% of total porosity). The CEC of Na–P1 and SZ was 28.80 and 24.40 meq/100 g, respectively. The share of micropores, mesopores, and macropores in Na–P1 was 13.3%, 71.6%, and 15.1%, while in the case of SZ it was 12.9%, 67.3%, and 19.8%, respectively. These parameters are important with regards to the sorption efficiency as they provide different channel sizes for organic compounds, metals, and gases, thus allowing or blocking the possibility of absorption.

**Figure 5:**
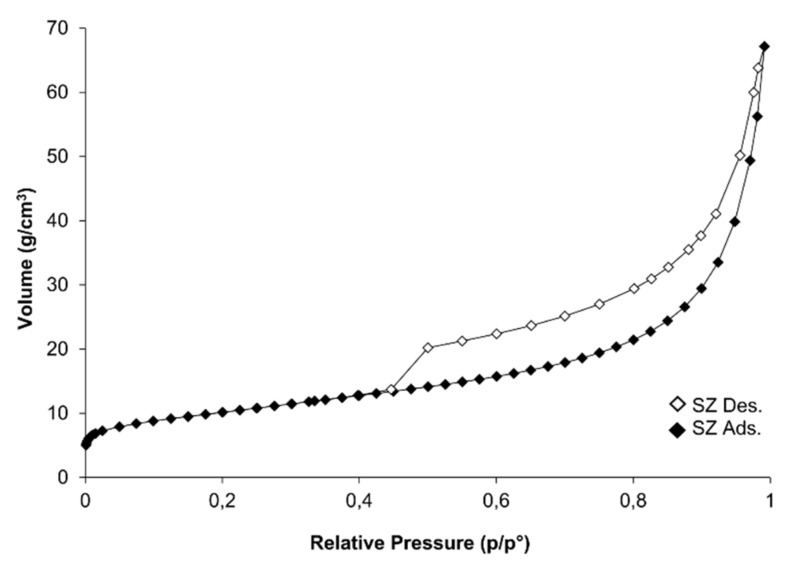
Comparison of N_2_ adsorption and desorption isotherms for the synthetic zeolite (SZ) sample.

The shape of N_2_ gas adsorption/desorption isotherms in the initial phase of p/p_0_ indicated type I. However, in the range of intermediate and higher pressures, it can be described as type II isotherm. Hysteresis loops were assigned as H2/H3 (according to IUPAC) (Figure 5). This type is most often characterized by pores occurring in the form of a slit or bottle. Such classification indicates the reversible filling of micropores driven by capillary condensation [51]. The shape of adsorption/desorption isotherms was typical for mesoporous materials.

**Figure 6:**
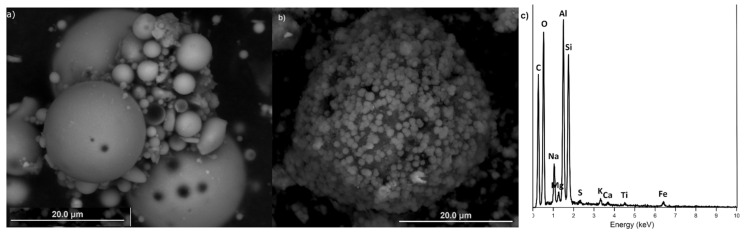
SEM images of (**a**) raw CFA and (**b**) zeolite SZ, (**c**) SEM-EDS results for SZ.4.3. Multi-element sorption experiments.

SEM observations of CFA used in the study clearly indicated its low quality. The grains were not spherical and appeared irregular (Figure 6a). However, the image in Figure 6b, shows the presence of zeolite structures and proves that the synthesis process was successful. The SEM observations of grain shapes showed that SZ consisted of isometric and octahedral crystals, approximately 2 μm in size, often intergrown with one another. In some areas, irregular aggregates were observed, as well. The SEM-EDS analysis of SZ grains visible in Figure 6b, showed the existence of mainly Al, O, Si, and C with minor amounts of Na and Mg (Figure 6c). This result further confirms that a zeolite phase was present in the SZ sample.

**Figure 7:**
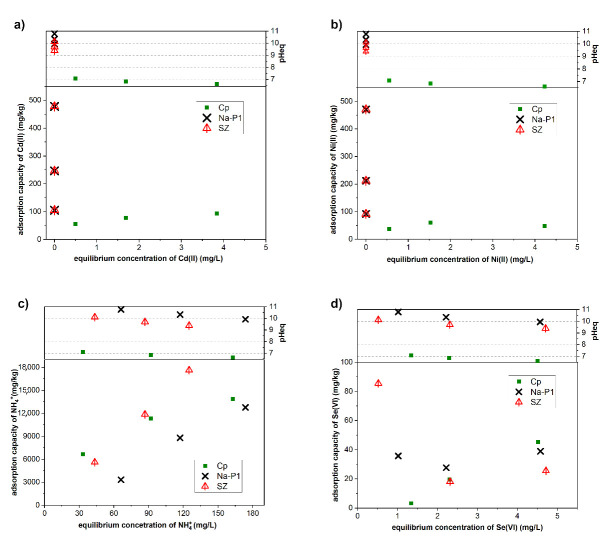
Sorption isotherms for (**a**) Cd(II); (**b**) Ni(II); (**c**) NH_4_^+^; and (**d**) Se(VI) on Cp, Na-P1, and SZ. The value above sorption isotherms indicates pH_eq_.

The sorption isotherms of SZ, Na–P1, and Cp for the adsorbed amounts of Cd(II), Ni(II), NH_4_^+^ cations, and Se(VI) anion are given in Figure 7. The multi-element sorption experiments revealed that the adsorption capacity of Cd(II) increased with the increasing C_0_ of Cd(II). However, in the case of Cp, only a slight increase in adsorption capacity was observed. The highest adsorbed amount for this sorbent was 93.73 mg/kg (at C_0_ of 5 mg/L). It can be attributed to the high C_0_ of NH_4_^+^ in solutions and consequently the competition of Cd(II) and NH_4_^+^ cations. In turn, SZ obtained in the experiment and Na–P1 achieved outstanding adsorption results. Cd(II) was removed from the solutions up to a concentration below the detection limit (<0.1 mg/L), regardless of C_0_. A similar result was observed for Na-P1. This can be linked to the high pH_eq_ of the solution which facilitates metal removal by the precipitation of hydroxides/oxyhydroxides. Maziarz et al. (2019) [52] reported that the precipitation of Cd(II) takes place at pH > 6.9. The significantly high removal of Ni(II) by the use of SZ and Na–P1 was also attributed to the precipitation of Ni(II) hydroxides/oxyhydroxides, which occurred at pH > 8 [53]. In the case of Cp, the adsorption capacity of Ni(II) increased with the increasing Ni(II) concentration and reached equilibrium, which is confirmed by the presence of plateau on adsorption isotherms. However, for this sorbent, the highest adsorbed amount of Ni(II) did not exceed 60 mg/kg.

In turn, the sorption results showed an increased uptake of NH_4_^+^ onto the tested zeolites with the increasing C_0_ of NH_4_^+^. This was associated with the increase in the C_0_ of NH_4_^+^ (from 100 to 300 mg/L) which leads to a greater driving force. As a consequence, at the highest initial concentration, NH_4_^+^ can efficiently migrate and exchange cationic ions (Na^+^, Ca^2+^) onto the external as well as internal surface of mesoporous zeolites [54]. Nevertheless, the SZ collected in the experiment presented the highest adsorption capacity among the zeolites analyzed in this study. For a C_0_ of 300 mg/L of NH_4_^+^, SZ absorbed 17,629 mg/kg, while Cp absorbed 13,854 mg/kg and Na–P1 absorbed 12,766 mg/kg. This suggests that SZ had a distinct affinity towards NH_4_^+^. The Si/Al ratio in the synthetic clinoptilolite, Na-P1 zeolite, and zeolite A is 4.84, 1.67, and 1.00, respectively [55]. Considering that the SZ sample is a mixture of Na-P1 zeolite and A zeolite, a simple relationship can be seen between the ammonium sorption size and the Si/Al ratio. The lower the Si/Al ratio, the greater the tendency to absorb ammonium by ion exchange. The greater amount of NH_4_^+^ sorption by Cp than by Na-P1 results from the admixture of clay minerals present in Cps natural sample.

In the case of Se(VI) removal, two different effects were observed. Cp exhibited an increased removal of Se(VI) with the increasing C_0_ of the element. However, the highest removed amount did not exceed 45.50 mg/kg (at C_0_ of 5 mg/L). Meanwhile, for SZ, a very good removal efficiency was achieved at Se(VI) C_0_ of 1 mg/L. At this concentration, the adsorption capacity reached 85.55 mg/kg. It is worth noticing that a similar but less significant effect was noticed for Na–P1. The removal of Se(VI) anions might also be associated with the precipitation of cadmium selenite (CdSeO_4_·2H_2_O) and nickel selenite (NiSeO_4_·6H_2_O). The low solubility product (K_sp_) of the potentially formed CdSeO_4_·2H_2_O (K_sp_ = 10^−1.85^) and NiSeO_4_·6H_2_O (K_sp_ = 10^−1.38^) [56] indicates the direction of equilibrium reaction towards the crystallization of low-soluble phases of selenite salts. In the control experiment Cd, Ni, Se, NH_4_^−^ were reacted according to the proportion: 5 mg Cd(II)/L + 5 mg Ni(II)/L + 5 mg Se(VI)/L + 300 mg NH_4_^+^/L with the pH adjusted to 7.13 (average value in the equilibrium solutions with Cp) and 10.15 (average value in the equilibrium solutions with Na-P1 and SZ). The reduction of Se(VI) initial concentration at pH 7.13 was 8.3% and at pH 10.15 was 9.5%. This is consistent with the results of Se(VI) sorption on the zeolite samples, where the reduction of the initial concentration of Se(VI) was: 9.1% as the result of the reaction with Cp and 9.8% in the reaction with SZ and Na-P1. On the other hand, the results of the analysis of Cd and Ni concentrations in the control experiment showed, depending on the pH and sorbents (7.13–Cp, 10.15–Na-P1, SZ), a reduction in the Cd concentration equal to 14% and 64%, and Ni 8% and 71%, respectively. In the sorption experiment, the reduction of the Cd concentration was 19% and 100%, and Ni 10% and 100%, respectively, for Cp, SZ, Na-P1. This clearly indicates the precipitation of cadmium selenite and nickel selenite as the main mechanism controlling the concentration of Se(VI). Ni and Cd can also precipitate as hydroxides, favoured by a pH of about 7 (Cp) and 10 (SZ, Na-P1). From the thermodynamic point of view, precipitation of cadmium selenite and nickel selenite may take place in the multi-element solutions. In this case, zeolite plays the role of a reagent removing metal ions and controlling the pH of the reaction.

The sorption of ions from the multi-element solution was a complex process due to the competing cation effect, which can significantly influence the uptake of Cd(II), Ni(II), and NH_4_^+^ onto the generally negatively charged surface of zeolites. The presence of Se(VI) anion also had a distinct impact on the sorption efficiency of all the ionic compounds present in the multi-element solution system. Nevertheless, the sorption experiments showed that the SZ sorption properties are comparable to or greater than those of natural Cp and synthetic Na–P1. Furthermore, the obtained SZ is capable of simultaneously removing cationic and anionic compounds. In the case of synthesis conducted with waste water NaOH solutions, the type of obtained zeolite phase was the same as with the SZ sample and the amount of zeolite phase was slightly lower. Due to this result, a similar sorption behavior with slightly lower efficiency is expected, taking into consideration the ions present already in the synthesis gel.

## 5. Conclusions

This study indicates the potential of using low-quality F-class CFA for the production of zeolites. Both NaOH_(DW)_ and NaOH_(WW)_ solutions were suitable for the zeolitization process at room temperature. Using wastewater rather than distilled water, slowed down the formation of the zeolite phase, but the observed effect was not strong enough to exclude the use of this medium in the process. During the experiments of zeolitization conducted in WFGD waste water, with 1 h of heating time in a temperature of 65 °C, zeolite X was observed in the samples. A temperature up to 65 °C at the primary stage of synthesis was the critical parameter. Depending on the synthesis parameters, the zeolite phase started to appear after 12 h to 3 weeks from the beginning of the experiment. Prolonging the heating time shortened the zeolitization process. Both XRD patterns and CEC value revealed that the amount of zeolite phase in the obtained material increased with the synthesis time. Based on the conducted measurements, the optimal synthesis conditions of the 12-h process duration with the 12-h heating time at 65 °C, were chosen. With those conditions, it was possible to obtain the mixture of Na-P1 and Zeolite A phase with the CEC value of 25.40 meq/100 g. The chemical analysis of the emerging material (SZ) showed a decrease of Si in favor of Al and Na. This indicated the transition of chemical composition from the initial CFA to the final zeolite phase. The quality of CFA used in the synthesis does not prevent the zeolitization process. In addition, the sample synthesized in the present study exhibited high sorption capabilities in comparison with the commercially available natural and synthetic zeolites. Thus, zeolites produced using the proposed technique could replace them as a cheaper alternative in technological applications that do not require materials of high purity. The examined parameters of the obtained zeolite material indicated that it might be useful as an adsorbent for various types of environmental pollutants.

The energy-efficient zeolitization process conditions, which are achievable in energy and heat production facilities, together with the use of waste heat and accessible on-site, low-quality CFA, and WFGD wastewater, can provide an energy- and environmentally-efficient method for producing functionalized materials. The obtained materials are characterized by promising performance parameters, thanks to the fact that they could be used at the very same power plants for the treatment of WFGD wastewater.

## Figures and Tables

**Table 1:** Chemical composition of coal fly ash (CFA1, CFA2), Clinoptilolite-Cp, and synthetic zeolite Na-P1.

[wt.%]	SiO_2_	TiO_2_	Al_2_O_3_	Fe_2_O_3_	CaO	MgO	K_2_O	Na_2_O	SO_3_	LOI	C
FA1	49.02	1.03	22.89	8.18	3.67	1.72	2.46	1.84	0.85	7.3	6.43
FA2	45.40	1.14	27.05	6.10	2.92	2.12	3.30	0.98	0.86	8.74	6.42
Cp	70.70	-	12.6	1.36	3.40	0.66	3.71	0.71	-	6.34	-
Na-P1	32.62	1.23	30.48	4.60	3.56	1.39	0.45	8.79	0.08	16.02	-

**Table 2:** Range and average of the most important parameters determined for the wet flue gas desulphurization (WFGD) wastewater streams used in this study.

		SO_4_^2−^	NO_3_^−^	NO_2_^−^	NH_4_^+^	Cl^−^
Range	mg/L	1287–1492	0.28–214	0.06–0.11	294–520	7445–8508
Average		1382	120.8	0.09	393	7976

**Table 3:** Zeolite synthesis experiment plan.

		Set 1	Set 2	Set 3	Set 4
First Stage		CFA + DW	CFA + WW	CFA + 3 M NaOH _(DW)_	CFA + 3 M NaOH _(WW)_
Second Stage	First Round	CFA + 3 M NaOH _(DW)_			
		T = 65 °C, t = 0 h	T = 65 °C, t = 1 h	T = 65 °C, t = 12 h	T = 65 °C, t = 24 h
	Second Round	Room temperature			
		CFA + 3 M NaOH _(DW)_	CFA + 4 M NaOH _(DW)_	CFA + 5 M NaOH _(DW)_	CFA + 6 M NaOH _(DW)_

**Table 4:** Summary of the textural parameters. Symbols: Cp—clinoptilolite (reference material); Na–P1—zeolite Na–P1 (reference material); and SZ—mixture of zeolite A and zeolite Na–P1 (synthesized in this study).

Material	*CEC* meq/100 g	*S_BET_* (m^2^/g)	$V_{tot}^{0.99}\;({\mathrm{cm}}^{3}/g)$	$V_{mik}^{DR}\;({\mathrm{cm}}^{3}/g)$	$V_{mez}^{BJH}\;({\mathrm{cm}}^{3}/g)$	$V_{mak}\;({\mathrm{cm}}^{3}/g)$
Cp	40.69	30.31	0.123	0.012	0.082	0.029
Na–P1	28.80	74.91	0.225	0.030	0.161	0.034
SZ	25.40	36.0	0.101	0.013	0.068	0.020

## Data Availability

The data presented in this study are available on request from the corresponding author.

## Abbreviations

CFA: F-class coal fly ash; deNO_x_: NO_x_ emission control installations; WFGD: Wet flue gas desulphurization installation; DW: Distilled water; WW: Wastewater from WFGD installation; Cp: Clinoptilolite-rich rock from the deposit in Nižný Hrabovec, Slovakia; Na–P1: Synthetic zeolite Na–P1 produced from F class fly ash in the hydrothermal conversion reaction; SZ: Synthetic zeolite, mixture of zeolite A and Na–P1, obtained after 12 h of synthesis with 3 M NaOH (DW) with 12 h heating time; C_0_: Initial Cd(II), Ni(II), Se(VI) or NH4+ concentration (mg/L); Ceq: The concentration of Cd(II), Ni(II), Se(VI) or NH4+ in the equilibrium solution (mg/L) after adsorption.
